# Stacking transgenic event DAS‐Ø15Ø7‐1 alters maize composition less than traditional breeding

**DOI:** 10.1111/pbi.12713

**Published:** 2017-04-11

**Authors:** Rod A. Herman, Brandon J. Fast, Peter N. Scherer, Alyssa M. Brune, Denise T. de Cerqueira, Barry W. Schafer, Ricardo D. Ekmay, George G. Harrigan, Greg A. Bradfisch

**Affiliations:** ^1^ Dow AgroSciences LLC Indianapolis IN USA; ^2^ Dow AgroSciencies Sementes e Biotecnologia Brasil LTDA Cravinhos SP Brazil; ^3^ Present address: The Coca‐Cola Company 1 Coca Cola Plaza Atlanta GA 30313 USA

**Keywords:** composition, breeding stacks, equivalence

## Abstract

The impact of crossing (‘stacking’) genetically modified (GM) events on maize‐grain biochemical composition was compared with the impact of generating nonGM hybrids. The compositional similarity of seven GM stacks containing event DAS‐Ø15Ø7‐1, and their matched nonGM near‐isogenic hybrids (iso‐hybrids) was compared with the compositional similarity of concurrently grown nonGM hybrids and these same iso‐hybrids. Scatter plots were used to visualize comparisons among hybrids and a coefficient of identity (per cent of variation explained by line of identity) was calculated to quantify the relationships within analyte profiles. The composition of GM breeding stacks was more similar to the composition of iso‐hybrids than was the composition of nonGM hybrids. NonGM breeding more strongly influenced crop composition than did transgenesis or stacking of GM events. These findings call into question the value of uniquely requiring composition studies for GM crops, especially for breeding stacks composed of GM events previously found to be compositionally normal.

## Introduction

Compositional analysis of genetically modified (GM) crops is universally required by regulatory authorities that assess safety. Compositional analysis was proposed more than 20 years ago to account for uncertainties in how the types of compositional changes and the magnitude of these changes might differ between crops that have been engineered to contain transgenes compared with those developed through traditional breeding (OECD, 1993). The variability in biochemical composition associated with nonGM crop varieties is not typically considered a significant safety risk (Bradford *et al*., 2005). Traditional breeding has a history of safe use, so the compositional variability among varieties developed through traditional breeding was the standard by which GM breeding techniques were to be assessed. After more than two decades of research, many published reports and hundreds of regulatory submissions, transgenesis has generally been found to have markedly less effect on crop composition compared with traditional breeding (Herman and Price, 2013). Advances in molecular biology have shown that the types of mutations that are possible during transgene insertion are similar to those associated with the intentional or unintentional random mutagenesis that occurs during traditional breeding, but that GM techniques typically have a smaller impact due to fewer genetic changes (Anderson *et al*., 2016; Harrigan *et al*., 2016; Schnell *et al*., 2015; Venkatesh *et al*., 2016).

While the potential for unintended compositional effects is now known to be markedly lower for GM crops compared with those developed using nonGM breeding techniques, government regulation and data requirements for GM crop composition have increased dramatically over the last 20 years, with a typical study now costing over one million US dollars (Herman and Price, 2013). Furthermore, when two or more previously approved GM events are crossed together (breeding stacks), many regulatory authorities require a new compositional study to be completed with equal complexity (and cost) to that required for novel transgenic events (EFSA Panel on Genetically Modified Organisms (GMO), 2011). Since transgenes are routinely incorporated into many new crop varieties after obtaining regulatory approvals, this requirement seems to be based on the premise that transgenes are more likely to interact with each other compared with interacting with the many different unique genotypes present in each new crop variety into which a GM event is introgressed.

### Purpose and approach

To provide a science‐based evaluation of the crop‐compositional effects of stacking GM events and to provide context around the regulatory requirement for composition studies with GM breeding stacks, we examined the grain composition of seven breeding stacks containing DAS‐Ø15Ø7‐1 maize (Baktavachalam *et al*., 2015) and other approved GM maize events. DAS‐Ø15Ø7‐1 expresses the Cry1F insecticidal protein and the phosphinothricin N‐acetyltransferase (PAT) herbicide‐tolerant enzyme. Event DAS‐Ø15Ø7‐1 has been crossed with other approved GM events expressing insecticidal and herbicide‐tolerance traits. These traits have known modes of action that are not expected to modify the endogenous metabolism of the plant and thus are not expected to alter crop composition.

Since current regulations in the European Union require the inclusion of commercial nonGM reference lines (or hybrids) in crop composition studies (EFSA Panel on Genetically Modified Organisms (GMO), 2011), we were able to evaluate the compositional differences between the GM breeding stacks and matched near‐isogenic nonGM hybrids (iso‐hybrids), and contrast that with the differences between the iso‐hybrids and nonGM reference hybrids. In this way, it was possible to directly compare the compositional effects of the multiple GM events in each breeding stack with compositional changes accompanying nonGM hybrid development.

## Results

### Purpose and context

The compositional profiles of nonGM near‐isogenic maize hybrids (iso‐hybrids) were compared with the same hybrids containing GM breeding stacks that included event DAS‐Ø15Ø7‐1 and were also compared with nonGM reference hybrids developed through traditional breeding. This approach allows the compositional effects of transgenesis, and the conventional crossing of GM events to be contrasted with non‐GM hybrid development techniques that are generally accepted as safe. Results are relevant to the scientific evaluation of government regulation for GM breeding stacks because such results reflect relative risk.

### Data analysis approach

While traditional statistical difference tests (e.g. ANOVA) have most commonly been used to compare crop composition between a GM variety and a nonGM near‐isogenic line (isoline), statistical differences observed using such approaches most commonly reflect the statistical power of the study rather than the effects of transgenesis. This is because the GM line is derived from a single cell from a single plant and does not reflect the intravarietal genetic variability of the line into which the GM trait was introgressed and with which it is most commonly compared (Fasoula and Boerma, 2007; Harrigan *et al*., 2016; Tokatlidis *et al*., 2008; Venkatesh *et al*., 2016). With sufficient statistical power, one would expect every compositional analyte to be found statistically different between the GM line and its isoline due to genetic differences that are not related to transgenesis, but rather due to endogenous genetic differences that are expected between two different single cell‐ or plant‐derived lines (intravarietal variation). Regulatory requirements for more and more complex and powerful experimental designs have led to greater and greater statistical detection of fleetingly small and biologically irrelevant compositional differences.

A complementary statistical approach has also been developed that is intended to represent the breadth of composition for nonGM varieties through construction of equivalence intervals based on a small number of reference lines included in the field trials, followed by determination of whether the composition for the GM line falls within these intervals (van der Voet *et al*., 2011; Ward *et al*., 2012). However, the conformation of the composition of the isoline (background genetics of GM line) to the average composition (or the compositional distribution) of the chosen reference lines may largely determine the results of such statistical tests. If the concentration of the measured compositional analyte in the isoline falls in the centre of the equivalence interval, then the GM line will likely also fall within the interval. The more distinct the isoline composition is from the average composition of the reference lines (or disparate from the compositional distribution of the reference lines), the more likely the composition of the GM line will be found to fall outside of the equivalence limits for the reference lines. That is, if the starting point for the isoline composition is in the centre of the equivalence interval for the reference lines, larger deviations from this composition will be seen as normal for the crop. Conversely, if the starting point for the isoline composition is near or outside of the equivalence limits for the reference lines, smaller deviations will often result in a finding of nonequivalence to the reference lines.

In summary, current statistical approaches to evaluating compositional equivalency between a GM line and the isoline using difference tests, or evaluating the compositional normalcy of the GM line by constructing equivalence limits based on a small number of nonGM reference lines, do not properly take into consideration intravarietal variability or the potential disparity of the composition of the isoline from the reference lines, respectively. In addition, multiplicity is most often not addressed in the simultaneous statistical evaluation of the many compositional endpoints under consideration, resulting in a high probability of declaring statistically significant differences where none exist (van der Voet *et al*., 2011). For these reasons, we chose to compare the iso‐hybrids in our analysis with both breeding stacks of DAS‐Ø15Ø7‐1 maize and with nonGM commercial hybrids using graphical methods intended to visualize the effects of transgenesis and GM breeding stacks on the profile of crop composition compared with non‐GM breeding. We quantified these compositional differences using an *I* ^2^ statistic (coefficient of identity) that estimates the fraction of the variation in the data captured by the line of identity.

### Focus is on transgenesis and stacking

We focused on GM entries that were treated with the same maintenance herbicides as the nonGM iso‐hybrids and the nonGM commercial reference hybrids. This avoided confounding any possible effects of transgenesis or stacking GM events with any possible effects of the trait‐associated herbicides, and because the use of herbicides on nonGM crop plants has a history of safe use. The application of herbicides to crops has never been associated with any adverse compositional change in a crop, and there is no reasonable hypothesis for why GM crops would respond differently to trait‐related herbicides (Herman and Price, 2013; Herman *et al*., 2013).

### Suitability of data for scatter plots

**Figure 1:**
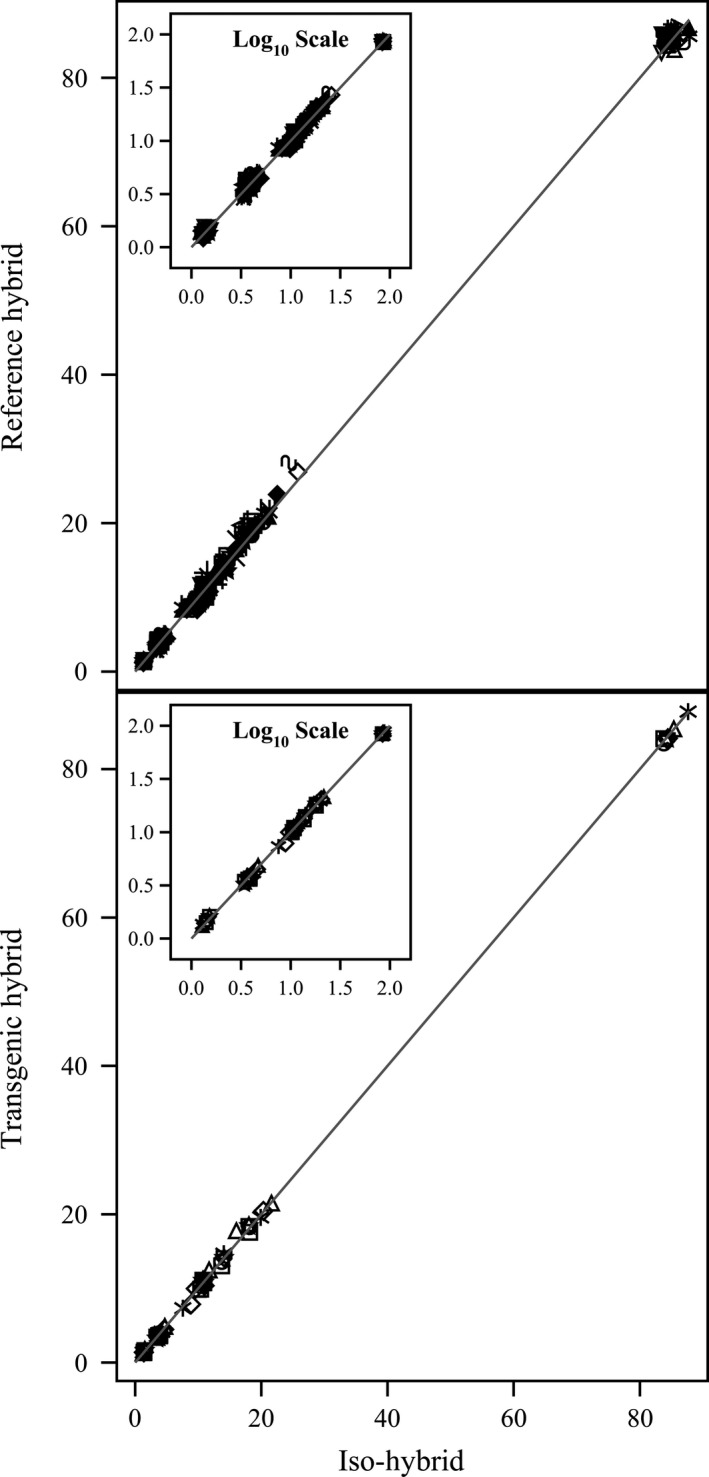
Proximates and fibre. Scatter plot of iso‐hybrids composition vs. location‐matched reference hybrids (upper panel) and DAS‐Ø15Ø7‐1 GM breeding stacks (lower panel). Analytes from left to right: ash, ADF, crude fat, NDF, crude protein, total dietary fibre, moisture and carbohydrates (moisture = %FW, all others = %DW). Line of identity (*y* = *x*) shown. The symbols representing each breeding stack and nonGM reference hybrid are listed in Table 1.

**Figure 2:**
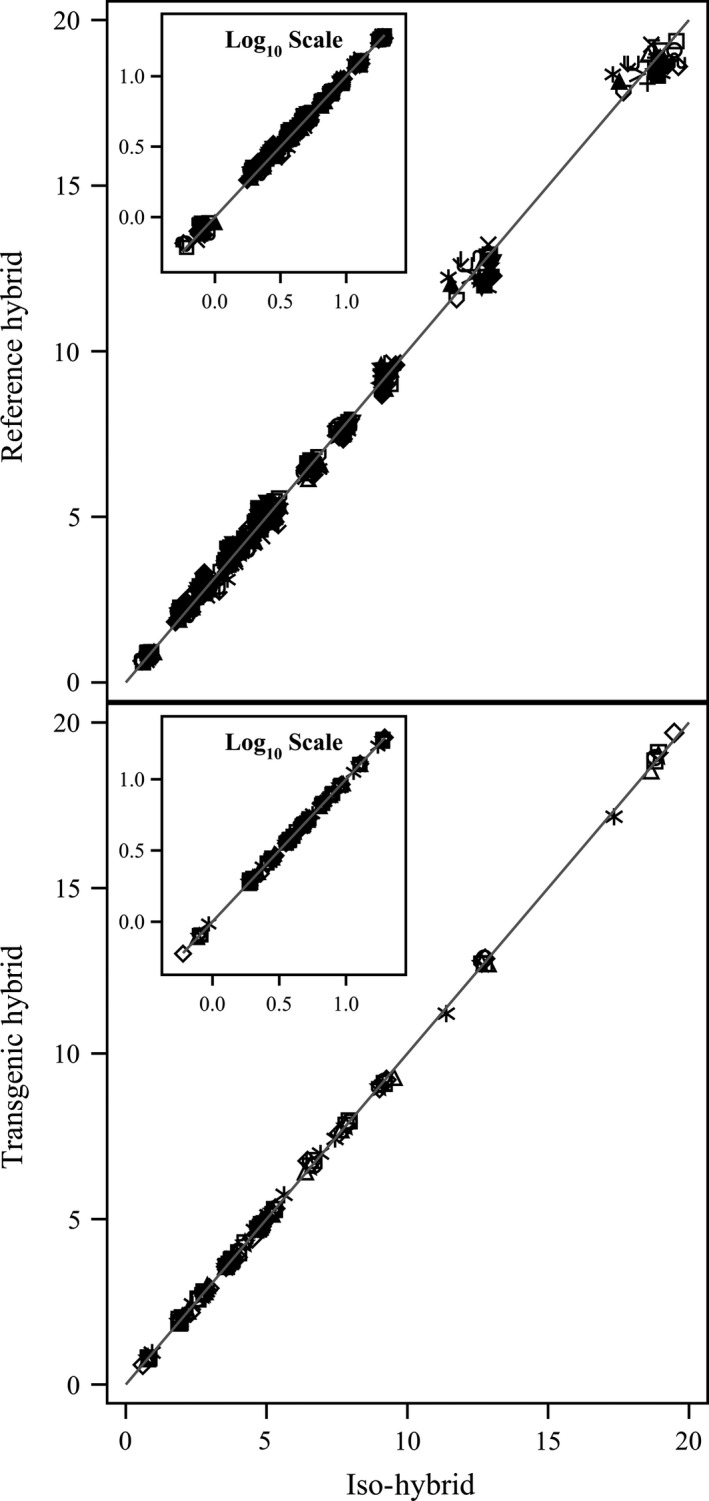
Amino acids. Scatter plot of iso‐hybrids composition vs. location‐matched reference hybrids (upper panel) and DAS‐Ø15Ø7‐1 GM breeding stacks (lower panel). Analytes from left to right: tryptophan, methionine, cystine, histidine, lysine, threonine, isoleucine, glycine, tyrosine, serine, valine, arginine, phenylalanine, aspartic acid, alanine, proline, leucine and glutamic acid (% of total amino acids). Line of identity (*y* = *x*) shown. The symbols representing each breeding stack and nonGM reference hybrid are listed in Table 1.

**Figure 3:**
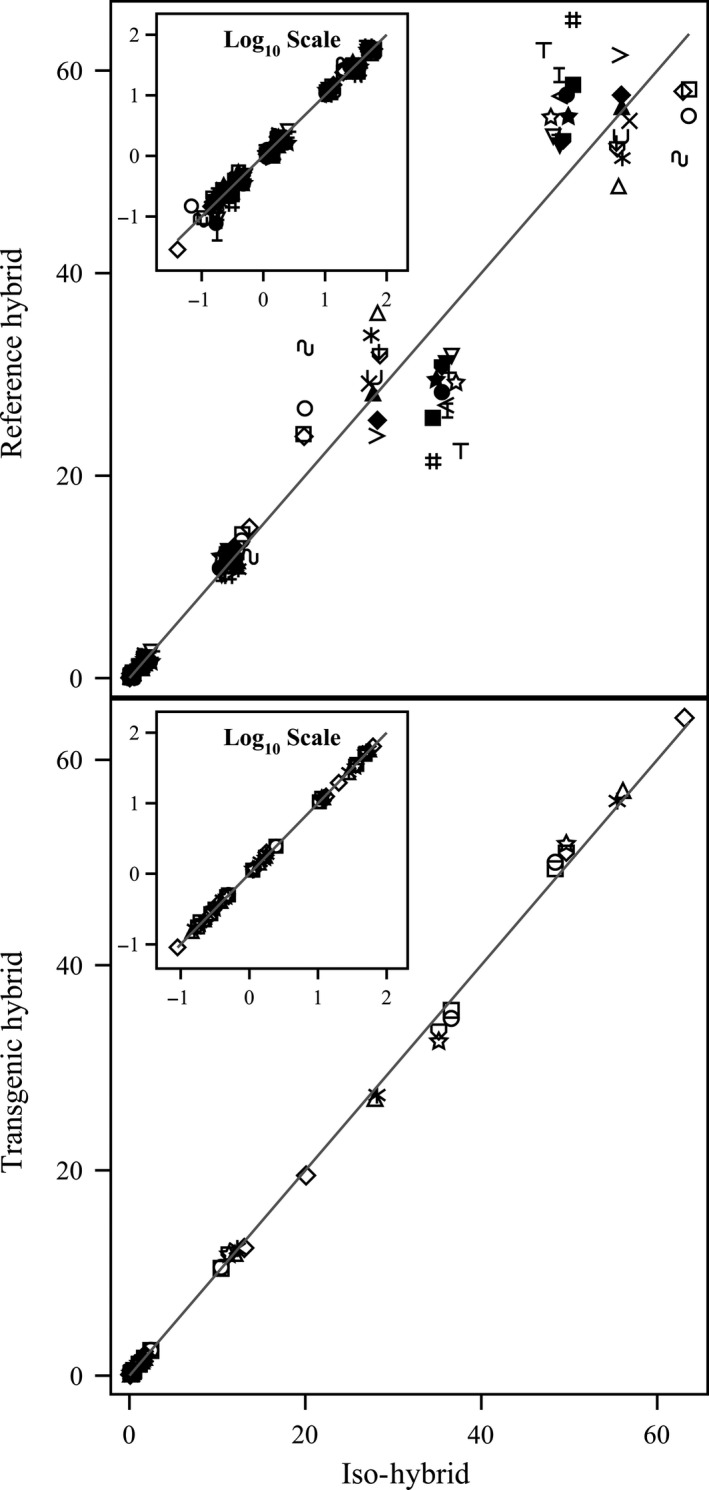
Fatty acids. Scatter plot of iso‐hybrids composition vs. location‐matched reference hybrids (upper panel) and DAS‐Ø15Ø7‐1 GM breeding stacks (lower panel). Analytes from left to right: 22 : 0 behenic, 20 : 1 eicosenoic, 20 : 0 arachidic, 18 : 3 linolenic, 18 : 0 stearic, 16 : 0 palmitic, 18 : 1 oleic and 18 : 2 linoleic (% of total fatty acids). Line of identity (*y* = *x*) shown. The symbols representing each breeding stack and nonGM reference hybrid are listed in Table 1.

**Figure 4:**
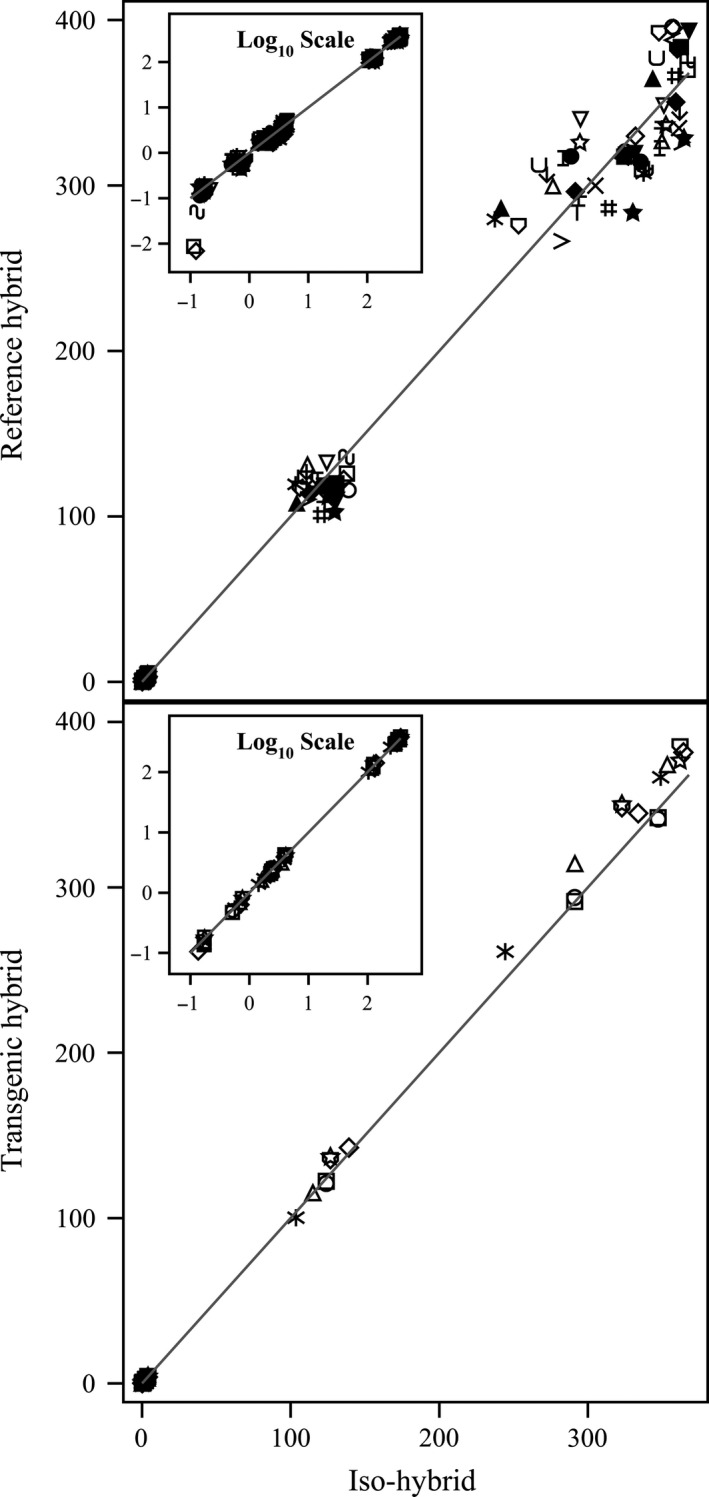
Minerals. Scatter plot of iso‐hybrids composition vs. location‐matched reference hybrids (upper panel) and DAS‐Ø15Ø7‐1 GM breeding stacks (lower panel). Analytes from left to right: copper, manganese, zinc, iron, calcium, magnesium, phosphorus and potassium (mg/100 g DW). Line of identity (*y* = *x*) shown. The symbols representing each breeding stack and nonGM reference hybrid are listed in Table 1.

**Figure 5:**
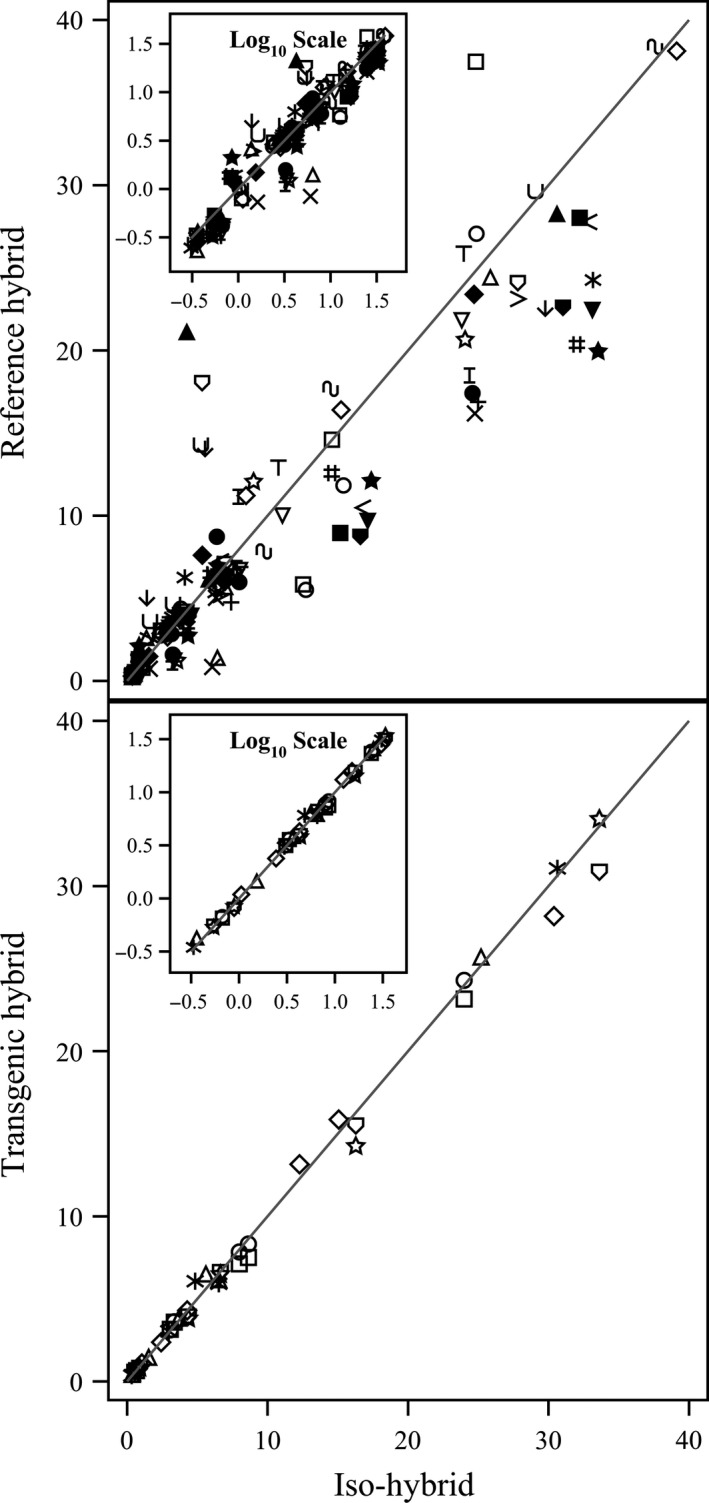
Vitamins. Scatter plot of iso‐hybrids composition vs. location‐matched reference hybrids (upper panel) and DAS‐Ø15Ø7‐1 GM breeding stacks (lower panel). Analytes from left to right: vitamin B_1_ (thiamine HCl), β‐carotene, vitamin B_6_ (pyridoxine HCl), α‐tocopherol and vitamin B_3_ (niacin) (mg/kg DW). Line of identity (*y* = *x*) shown. The symbols representing each breeding stack and nonGM reference hybrid are listed in Table 1.

**Figure 6:**
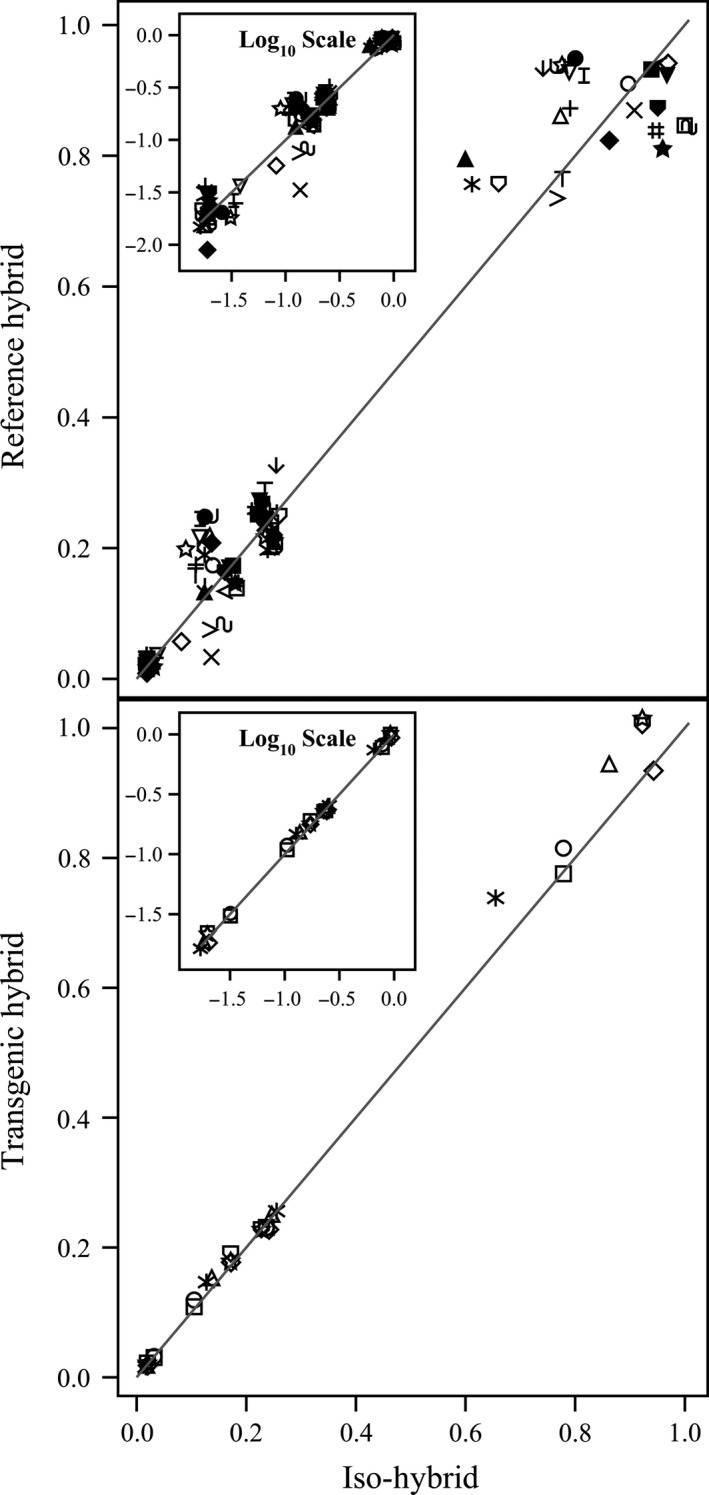
Secondary metabolites. Scatter plot of iso‐hybrids composition vs. location‐matched reference hybrids (upper panel) and DAS‐Ø15Ø7‐1 GM breeding stacks (lower panel). Analytes from left to right: *p*‐coumaric acid, raffinose, ferulic acid and phytic acid (%DW). Line of identity (*y* = *x*) shown. The symbols representing each breeding stack and nonGM reference hybrid are listed in Table 1.

Previous analyses of soybean using six categories of analytes illustrated a reasonable spread of analyte concentrations along the regression lines and a subsequently adequate depiction of profiles within each analyte category (Fast *et al*., 2016). A similar situation was observed here for maize (Figures 1, 2, 3, 4, 5, 6). This approach allows a more contextual interpretation of results by not isolating single analytes from related compositional components. This approach also weights more predominant analytes more heavily than less predominant analytes in calculating the *I* ^2^ statistic and does not inflate small absolute concentration changes for low‐prevalence analytes by expressing them as a per cent of the isoline concentration. In the absence of a hypothesis for a GM trait causing a specific compositional change, this procedure seems reasonable for assessing unintended compositional changes.

To investigate the potential effects of lower prevalence compositional analytes on observed trends, the data were also transformed to the base‐10 logarithm which weights smaller values more heavily than is the case in the natural scale. These data were plotted and used to generate *I* ^2^ statistics to supplement the analysis in the natural scale but are not discussed in detail (Table 1; Figures 1, 2, 3, 4, 5, 6 insets). For some analyte profiles, data expressed as the base‐10 logarithm are more evenly spread along the line of identity (e.g. proximates and fibre, Figure 1), but it is noteworthy that, unlike regression lines, the line of identity is fixed, and its position is not influenced by compositional deviations from the line.

**Table 1:**
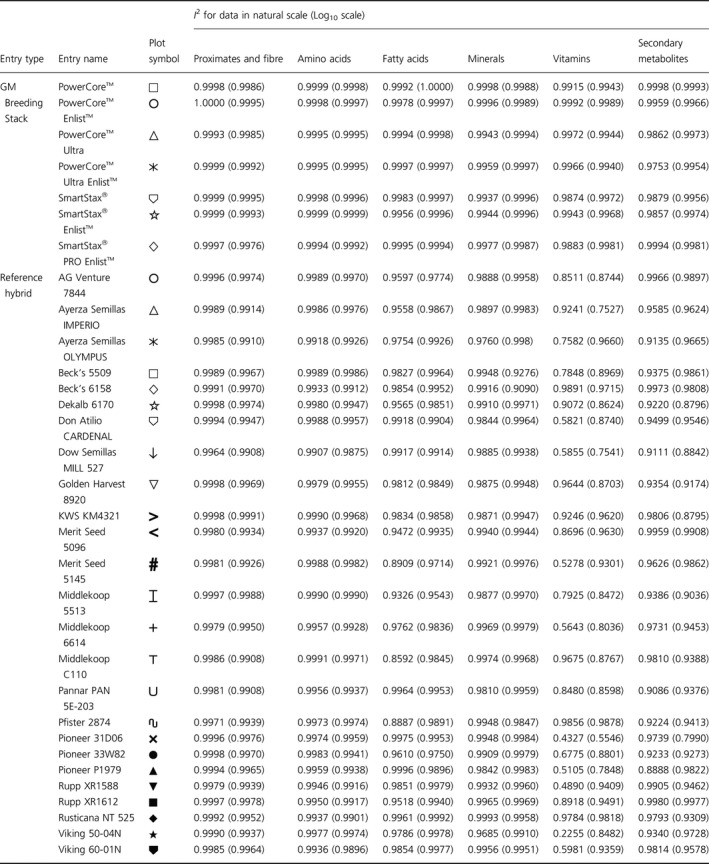
Plot symbols and coefficients of identity (*I* ^2^) for iso‐hybrids predicting indicated hybrid composition

### NonGM reference line composition

As expected, the line of identity (*y* = *x*) approximated the relationship between the iso‐hybrids and reference hybrids well with the exception of vitamins for a subset of the reference hybrids; vitamins in maize are known to vary widely (Figures 1, 2, 3, 4, 5, 6, upper panels; Table 1; Lundry *et al*., 2013). Maize grain is typically sold as a generic commodity without price adjustments based on compositional variation (with the exception of high moisture which is docked to account for required drying to preserve quality). This practice is only practical if a reasonably consistent nutrient composition is observed. The *I* ^2^ values for the iso‐hybrids predicting the twenty‐five reference hybrids are ≥0.9964 for proximates and fibre, ≥0.9907 for amino acids, ≥0.8592 for fatty acids, ≥0.9685 for minerals, from 0.2255 to 0.9891 for vitamins and ≥0.8888 for secondary metabolites (Table 1).

### DAS‐Ø15Ø7‐1 maize breeding stack composition

Also as expected, the line of identity approximated the relationship between the iso‐hybrids and the DAS‐Ø15Ø7‐1 maize breeding stacks extremely well for all analyte profiles (Figures 1, 2, 3, 4, 5, 6, lower panels; Table 1). The insertion of GM traits that are not expected to affect endogenous metabolic pathways (as is the case here), and their cross‐breeding into stacks is not expected to alter the composition of a crop as much as the recombination of many thousands of genes during traditional nonGM breeding. The *I* ^2^ values for the iso‐hybrids predicting the seven matched DAS‐Ø15Ø7‐1 maize breeding stacks are ≥0.9993 for proximates and fibre, ≥0.9994 for amino acids, ≥0.9956 for fatty acids, ≥0.9937 for minerals, ≥0.9874 for vitamins and ≥0.9753 for secondary metabolites (Table 1).

### GM breeding stack vs. traditional breeding

**Figure 7:**
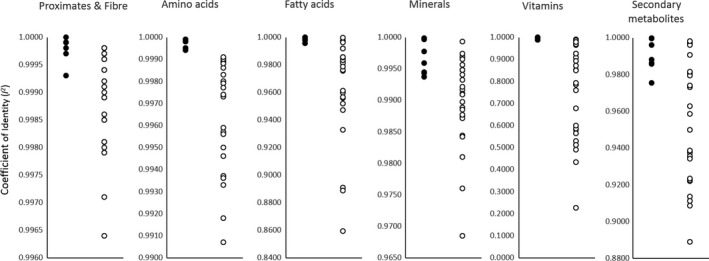
Coefficient of identity (*I* ^2^) for iso‐hybrids predicting GM breeding stacks (solid circles) and nonGM commercial reference hybrids (open circles) for indicated compositional analyte profile.

Our data analysis approach allows the direct comparison between traditional breeding and transgenic approaches including stacking GM traits by traditional breeding (breeding stacks). We used the nonGM iso‐hybrids as a calibrator against which these breeding techniques were evaluated. Several weaknesses of statistical difference tests and equivalence tests were avoided, including the isolation of one analyte from related analytes (avoided through examination of compositional profiles). Both graphically and through the statistical estimation of compositional identity (*I* ^2^), the greater influence of traditional breeding on composition, compared with transgenesis and stacking GM traits by traditional breeding, is clearly evident (Table 1, Figures 1, 2, 3, 4, 5, 6). Transformation of composition values to the base‐10 logarithmic scale (weighting lower prevalence analytes more heavily than in the natural scale) does not change this conclusion (Table 1, Figures 1, 2, 3, 4, 5, 6 insets). A comparison of the distributions of *I* ^2^ values for the iso‐hybrids predicting the DAS‐Ø15Ø7‐1 maize breeding stacks vs. the nonGM commercial reference lines clearly indicates greater compositional changes associated with traditional breeding versus stacking GM events (Figure 7).

## Discussion

Over 46 000 compositional data points are summarized here in a relatively small number of informative figures and tables. The results from this analysis are consistent with previous reports that transgenesis has a negligible influence on crop composition when traits are not expected to alter the endogenous metabolism of plants (Herman and Price, 2013). Specifically, there is extremely weak justification for requiring special compositional testing of GM breeding stacks where the component events have been found compositionally equivalent to the nonGM crop (Kok *et al*., 2014; Pilacinski *et al*., 2011). The use of difference and equivalence testing to analyse GM compositional equivalence does not adequately account for intravarietal variation, or the potential disparity of the composition of the isoline from the reference lines, respectively, nor is multiplicity typically addressed in analyses using these statistical approaches. The use of scatter plots and the estimation of compositional identity (*I* ^2^) for the isoline versus the GM lines and the reference lines allows the potential effects and risks of altering crop composition due to transgenesis to be put into context. Without putting the risks of transgenesis and stacking of GM traits into the context of traditional breeding and grower practices, regulatory authorities risk diverting limited resources to evaluating negligible risks while leaving appreciable risks uncharacterized (Buchholz *et al*., 2011). Scientific evidence strongly supports a proportionally low level of regulatory burden for GM breeding stacks based on their very low risk.

## Experimental procedures

### Field trials

Five field studies were conducted between 2010 and 2016 across two countries (USA and Argentina) with a total of seven DAS‐Ø15Ø7‐1 maize breeding stacks. The GM events in each breeding stack, the locations of the trials and the nonGM reference hybrids included at each location are listed in Table 2. The gene products expressed in the breeding stacks are listed in Table 3.

**Table 2:** Field trial information

	Breeding stacks in indicated study				
GM event	Study 101061	Study 101062	Study 141098	Study 150126	Study 151077
DAS‐Ø15Ø7‐1	X X	X X	X	X	X
DAS‐59122‐7	X X			X	
DAS‐4Ø278‐9	X	X		X	X
SYN‐IR162‐4			X		X
MON‐89Ø34‐3	X X	X X	X	X	X
MON‐ØØ6Ø3‐6		X X	X		X
MON‐88Ø17	X X				
MON‐87427‐7				X	
MON‐87411				X	
Commercial Name	SmartStax^®^ & SmartStax^®^ Enlist™	PowerCore™ & PowerCore™ Enlist™	PowerCore™ Ultra	SmartStax^®^ PRO Enlist™	PowerCore™ Ultra Enlist™

Growing season	2010	2010	2014/2015	2015	2015/2016
Country	USA	USA	Argentina	USA	Argentina
Site 1	IA, Richland	IA, Richland	Buenos Aires, San Pedro	IA, Kimballton	Santa Fé, Amenábar
Site 2	IA, Jefferson	IA, Lime Springs	Buenos Aires, Berdier	IA, Cresco	Buenos Aires, Berdier
Site 3	IA, Lime Springs	IA, Atlantic	Buenos Aires, Carmen de Areco	IA, Richland	Buenos Aires, Blandengues
Site 4	IA, Atlantic	IL, Carlyle	Buenos Aires, Inés Indart	IL, Stewardson	Buenos Aires, Chacabuco
Site 5	IL, Wyoming	IL, Wyoming	Buenos Aires, El Crisol	IN, Pickard	Buenos Aires, Capitán Sarmiento
Site 6	MI, Deerfield	IN, Sheridan	Buenos Aires, Los Indos	MO, Kirkville	Córdoba, Mattaldi
Site 7	MN, Cherry Grove	MO, Fisk	Buenos Aires, San Patrico	NE, York	Buenos Aires, Tacuarí
Site 8	MN, Geneva	NE, Brunswick	Buenos Aires, Tacuarí	PA, Germansville	Santa Fé, Villa Cañás
Site 9	NE, Brunswick	NE, York	–	–	–
Site 10	PA, Germansville	PA, Germansville	–	–	–

	Reference hybrids^a^ (planting sites)				
Reference 1	Viking 60‐01N (1, 2, 4, 6, 10)	Golden Harvest 8920 (1, 4, 7, 9, 10)	Pannar PAN 5E‐203 (1, 2, 5)	Pfister 2874 (1, 5, 6)	Pioneer P1979 (1, 6, 7)
Reference 2	Viking 50‐04N (1, 3, 4, 5, 7, 8)	Middlekoop 6614 (1, 2, 3, 8, 9)	KWS KM4321 (2, 6, 7, 8)	Beck's 5509 (1, 2, 7)	Ayerza Semillas OLYMPUS (2, 4, 6, 8)
Reference 3	Rupp XR1588 (1, 5, 9, 10)	Middlekoop C110 (1, 3, 5, 6, 7)	Pioneer 31D06 (1, 2, 3, 4, 6)	Beck's 6158 (1, 3, 6)	Ayerza Semillas IMPERIO (5, 8)
Reference 4	Rupp XR1612 (2, 3, 7, 8, 10)	Middlekoop 5513 (2, 6, 7, 8, 10)	Dow Semillas MILL 527 (3, 4, 7)	*Becks 6175 (6, 8)*	Don Atilio CARDENAL (4, 5, 7)
Reference 5	Merit Seed 5096 (2, 5, 6, 8, 9)	Pioneer 33W82 (2, 3, 4, 5, 10)	Ayerza Semillas IMPERIO (1, 4, 5, 7, 8)	*Beck's 5828 (7)*	Dow Semillas MILL527 (1, 2, 5)
Reference 6	Merit Seed 5145 (3, 4, 6, 7, 9)	Dekalb 6170 (4, 5, 6, 8, 9)	Rusticana NT 525 (3, 5, 6, 8)	*Beck's 6272 (7)*	KWS KM4321 (2, 3, 7)
Reference 7	–	–	–	AG Venture 7844 (2, 3, 4)	*Rusticana NT 525 (6)*
Reference 8	–	–	–	*Master's Choice MC6060 (2, 8)*	*Rusticana NT 426 (3, 4)*
Reference 9	–	–	–	*Master's Choice MC6150 (4)*	Pannar PAN 5E‐203 (1, 3, 8)
Reference 10	–	–	–	*Viking 52‐11N (3, 5)*	–
Reference 11	–	–	–	*Mycogen 2H721 (4, 5)*	–
Reference 12	–	–	–	*Dairyland DS‐1811 (8)*	–

a Reference hybrids in italics were represented at fewer than three field sites and were therefore excluded from analysis.

**Table 3:** Transgenic gene products

GM event	Insect protection	Herbicide tolerance
DAS‐Ø15Ø7‐1	Cry1F	PAT
DAS‐59122‐7	Cry34Ab1, Cry35Ab1	PAT
DAS‐4Ø278‐9	–	AAD‐1
SYN‐IR162‐4	Vip3Aa20	–
MON‐89Ø34‐3	Cry1A.105, Cry2Ab2	–
MON‐ØØ6Ø3‐6	–	CP4 EPSPS
MON‐88Ø17	Cry3Bb1	CP4 EPSPS
MON‐87427‐7	–	CP4 EPSPS
MON‐87411	Cry3Bb1, DVsnf7	CP4 EPSPS

Plots were arranged in a randomized complete block design with four blocks at each location. Three commercial nonGM reference hybrids were planted at each location, and the reference hybrids (≥6 per study) were distributed randomly among locations. Plots at each location were 2‐4 rows wide, and each plot was bordered by two rows of nonGM maize. Field sites were surrounded by a maize border of at least four rows. Blocks were separated by at least 1.5 m of bare soil. Seed was planted between 15 and 25 cm apart in the row. Plots were between 6 and 10 m long with row spacing ranging from 70 to 76 cm. Appropriate agronomic practices were implemented at each field site across all plots to produce a commercially acceptable crop. Grain samples were randomly collected at physiological maturity and consisted of approximately 500 g (approximately 5 ears) that were stored frozen until analysis.

### Grain analysis

Compositional analyses of maize grain samples focused on six key analyte categories (proximates and fibre, amino acids, fatty acids, minerals, vitamins and secondary metabolites; OECD, 2002). Methods of compositional analysis have been previously reported (Herman *et al*., 2010). Analytes within each category are listed in the figure captions (Figures 1, 2, 3, 4, 5, 6).

### Data analysis and interpretation

For each of the six categories of analytes, a scatter plot of each mean analyte level (across locations) for each DAS‐Ø15Ø7‐1 breeding stack was plotted against the corresponding mean iso‐hybrid analyte level such that a finding of identical composition would result in points falling on the line of identity (*y* = *x*). Each GM breeding stack was represented by a unique symbol on these plots (Table 1). Plots were also generated where the mean analyte levels for each nonGM commercial reference hybrid were plotted against the mean levels of the iso‐hybrid across the same sites as those where that reference hybrid was grown. Only reference hybrids grown at three or more sites were included in the analysis. For the four nonGM reference hybrids included in both Argentine studies (Table 2), means were calculated across both studies. The level of data scatter around the line of identity was used to compare the compositional effects of transgenesis and stacking with that of nonGM breeding.

To quantify the level of compositional identity between the iso‐hybrid and each GM breeding stack or non‐GM reference hybrid, the amount of data variation accounted for by the line of identity was calculated (defined as coefficient of identity or *I* ^2^). The *I* ^2^ statistic was calculated as for the coefficient of determination (*R* ^2^) except that the measured *x* value (*x*_*i*_) was substituted for the predicted *y* value (*ŷ*_*i*_) in the following equation.

$$R^{2}=1-\frac{\sum_{i=1}^{N}{\left(y_{i}-\hat{y}\right)}^{2}}{\sum_{i=1}^{N}{\left(y_{i}-\overset{¯}{y}\right)}^{2}}$$

This statistic estimates the fraction of the variability in the data that is accounted for by the line of identity.

$$I^{2}=1-\frac{\sum_{i=1}^{N}{\left(y_{i}-x_{i}\right)}^{2}}{\sum_{i=1}^{N}{\left(y_{i}-\overset{¯}{y}\right)}^{2}}$$

Plots and *I* ^2^ statistics were generated for composition values in both the natural scale and for data transformed to the base‐10 logarithmic scale.

## Author contributions

All authors contributed to the writing of the text. RH drafted initial text and conceived of the data analysis approach. GB led the effort to publish composition results for GM breeding stacks. BF performed data analysis and production of figures. PS modified the coefficient of determination equation to calculate the coefficient of identity. BF, AB and DdC oversaw field trials under the direction of BS. RE and GH contributed to the text placing study results into the context of traditional breeding.

## Conflict of interest

All authors currently or formerly are/were employed by companies that develop and market transgenic seed.
